# Thermophilic Chloroflexi Dominate in the Microbial Community Associated with Coal-Fire Gas Vents in the Kuznetsk Coal Basin, Russia

**DOI:** 10.3390/microorganisms9050948

**Published:** 2021-04-28

**Authors:** Vitaly V. Kadnikov, Andrey V. Mardanov, Alexey V. Beletsky, Mikhail A. Grigoriev, Olga V. Karnachuk, Nikolai V. Ravin

**Affiliations:** 1 Institute of Bioengineering, Research Center of Biotechnology of the Russian Academy of Sciences, 119071 Moscow, Russia; vkadnikov@bk.ru (V.V.K.); mardanov@biengi.ac.ru (A.V.M.); mortu@yandex.ru (A.V.B.); 2 Laboratory of Biochemistry and Molecular Biology, Tomsk State University, 634050 Tomsk, Russia; plkmnt101@mail.ru (M.A.G.); olga.karnachuk@green.tsu.ru (O.V.K.)

**Keywords:** thermophiles, coal gases, hydrogenotrophs, microbial community, Chloroflexi, *Candidatus* Udaeobacter

## Abstract

Thermal ecosystems associated with areas of underground burning coal seams are rare and poorly understood in comparison with geothermal objects. We studied the microbial communities associated with gas vents from the coal-fire in the mining wastes in the Kemerovo region of the Russian Federation. The temperature of the ground heated by the hot coal gases and steam coming out to the surface was 58 °C. Analysis of the composition of microbial communities revealed the dominance of *Ktedonobacteria* (the phylum Chloroflexi), known to be capable of oxidizing hydrogen and carbon monoxide. Thermophilic hydrogenotrophic Firmicutes constituted a minor part of the community. Among the well-known thermophiles, members of the phyla Aquificae, Deinococcus-Thermus and Bacteroidetes were also found. In the upper ground layer, Acidobacteria, Verrucomicrobia, Actinobacteria, Planctomycetes, as well as Proteobacteria of the alpha and gamma classes, typical of soils, were detected; their relative abundancies decreased with depth. The phylum Verrucomicrobia was dominated by *Candidatus* Udaeobacter, aerobic heterotrophs capable of generating energy through the oxidation of hydrogen present in the atmosphere in trace amounts. Archaea made up a small part of the communities and were represented by thermophilic ammonium-oxidizers. Overall, the community was dominated by bacteria, whose cultivated relatives are able to obtain energy through the oxidation of the main components of coal gases, hydrogen and carbon monoxide, under aerobic conditions.

## 1. Introduction

Studies of thermophilic microorganisms that survive and develop at extreme temperatures for ordinary life have expanded our understanding of the diversity of microorganisms, their evolution, and mechanisms of adaptation to extreme environmental conditions [1,2]. Most research on thermophiles has focused on thermal ecosystems associated with volcanic activity, such as hot springs and deep-sea hydrothermal vents, or biotechnologically important objects, such as high-temperature bioreactors, etc. In addition to volcanic activity, the natural combustion of fossil hydrocarbons and coal can lead to the formation of thermal ecological niches. The phenomena of underground burning coal seams are quite common in nature and are found in Australia, Germany, USA (Pennsylvania), China, Russia, India and other countries [3]. Such underground fires can last for centuries, for example, a coal seam in Dudweiler (Saar, Germany) has been burning since 1668. An example of long-term natural underground coal fire is Burning Mountain in Australia [4], the burning time of which is estimated to be approximately 6000 years old.

Combustion of coal under low oxygen conditions and the presence of water is accompanied by the formation of coal gases in the reaction 3 C + O_2_ + H_2_O → H_2_ + 3 CO [5]. Molecular hydrogen can also be formed in a water gas shift reaction: CO + H_2_O → CO_2_ + H_2_. Underground coal fire is a natural analogue of the synthesis gas production process during coal gasification. Gases emanating to the surface can also contain light hydrocarbons, hydrogen sulfide, sulfur oxides, and other toxic compounds such as benzene, xylene, aliphatic and halogenated compounds [3,6,7]. Sulfur compounds and other elements found in coal seams can be delivered to the surface by the gas stream, resulting in contamination of the surrounding areas [8]. The temperature of the gas coming out to the surface depends on the temperature at which the underground coal fire takes place and on the depth of the fire, and is usually in the range from 50 to 800 °C.

In areas where hot coal gases come to the surface, local extreme ecosystems characterized by high temperatures (>50 °C) and the presence of toxic substances were formed [9]. Hydrogen and CO contained in coal gases can be used by microorganisms as high-energy substrates, which determines the possibility of the development of specific communities of thermophilic microorganisms. However, little is known about the composition of microbial communities in such ecosystems.

The Kuznetsk coal basin (Kemerovo region, Russia) is one of the largest in the world [10]. Coal mining in this region began in the middle of the 18th century. Initially, mining was carried out by the mine method, but recently many mines have been closed. At the moment, coal is mined in an open way, using overburden operations. The dumps formed after mining can periodically ignite when the incoming oxygen from the surface and coal interact [11]. At present, in the area of the city of Kiselevsk, a large number of spontaneous fire areas have been recorded in coal deposits.

The aim of this study was to uncover the composition of microbial communities associated with an underground coal fire site near the city of Kiselevsk, Kemerovo Region, Russia, using 16S rRNA gene sequencing.

## 2. Materials and Methods

### 2.1. Study Site, Sampling, and Chemical Analyses

**Figure 1:**
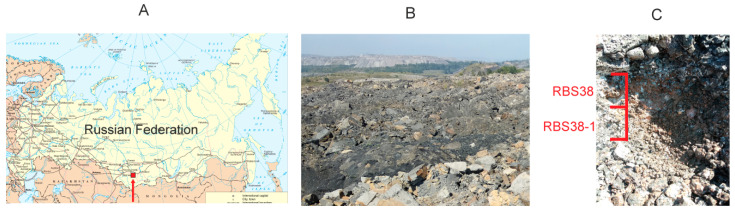
The site of underground coal fire studied in the work. (**A**) Location map. The red rectangle shows the location of the study area. (**B**) General view of the area. (**C**) Sampling site.

Samples were taken at an abandoned open coal pit near the town of Kiselevsk, Kemerovo Region (Figure 1). At the moment, within the city limits, 9 open-cut coal mines are in operation, and all underground mines are closed. At one of the quarries, located near the Dalnie Gory mine, signs of an underground fire were found in the forms of intense heating of the ground and the rise of clouds of steam with smoke.

Ground samples were taken with a hand shovel on August 8, 2020 from a dump of a coal mine in the area of smoke and hot steam coming out to the surface (54°02′06.3″ N 86°36′52.8″ E). The first sample, designated RBS38, was taken from the ground surface (0–5 cm depth). The second sample, RBS38-1, was taken from the same point in the ground at a depth of 5–10 cm. The samples (~30 g each) were collected in sterile plastic tubes with a volume of 50 mL. All samples were stored at + 4 °C prior to analysis.

To determine the composition of ions, 10 mL of deionized water was added to 14 g of the RBS38 sample. The mixture was incubated for 1 h with periodic stirring, then the liquid was taken and passed through a filter with a pore diameter of 0.22 μm (Merck Millipore, Burlington, MA, USA). The concentrations of nitrate, sulfate, fluoride, and chloride in the filtered liquid were determined by ion chromatography using a Stayer high performance liquid chromatographic gradient system (Aquilon, Moscow, Russia) with Dionex™ IonPac™ AS22IC column (Thermo Fisher Scientific, Waltham, MA, USA).

### 2.2. Mineralogical and Elemental Analysis

The mineralogical composition and elemental composition of RBS38 sample were characterized by X-ray diffraction (XRD) on a Shimadzu XRD-6000 diffractometer (Shimadzu Corporation, Kyoto, Japan) and by scanning electron microscopy (SEM) on a Philips SEM 515 (Philips Electronic Instruments) with energy dispersive spectrometry (EDS; EDAX Inc., Mahwah, NJ, USA) as previously described [12].

### 2.3. 16S rRNA Gene Sequencing and Analysis

Total DNA samples were isolated from 3 g of ground using a DNeasy PowerMax Soil Kit (Qiagen). PCR fragments of the 16S rRNA gene were amplified from total DNA using universal primers 341F (5′-CCTAYGGGDBGCWSCAG-3′) and 806R (5′-GGACTACNVGGGTHTCTAAT-3′) [13]. PCR fragments were barcoded using the Nextera XT Index Kit v. 2 (Illumina, San Diego, CA, USA) and purified using Agencourt AMPure beads (Beckman Coulter, Brea, CA, USA). The concentration of the resulting PCR products was determined using a Qubit dsDNA HS Assay Kit (Invitrogen, Carlsbad, CA, USA). Then PCR fragments were mixed in equimolar amounts and sequenced on Illumina MiSeq (2 × 300 nt paired-end reads). Paired overlapping reads were merged using FLASH v.1.2.11 [14]. The resulting sequences were clustered into operational taxonomic units (OTUs) at 97% identity using the Usearch software [15]; low quality reads, chimeric sequences, and singletons were removed by clustering.

Taxonomic assignment of OTUs was performed by searches against the SILVA v.132 database using the VSEARCH algorithm [16]. To calculate OTUs relative abundances, all reads (including singleton and low-quality reads) were mapped to OTU sequences at 97% global identity threshold by Usearch.

For phylogenetic analysis the OTU sequences were aligned using MAFFT v.7.310 [17]. The maximum likelihood phylogenetic tree was computed by FastTree v.2.1.3 [18]. Bootstrap tests were performed with 1000 resamplings. The default settings were used for all software unless otherwise noted.

### 2.4. Nucleotide Sequence Accession Numbers

The raw data generated from 16S rRNA gene sequencing have been deposited in the NCBI Sequence Read Archive (SRA) under the accession numbers SRX10002666 (sample RBS38) and SRX10002667 (sample RBS38-1) as part of the BioProject PRJNA697856.

## 3. Results and Discussion

### 3.1. Physicochemical and Mineralogical Characteristics of Ground Samples

Ground samples were taken at an abandoned open coal pit in the area of underground coal fire. The first sample, RBS38, was taken from the surface to a depth of 5 cm, whiles the second sample, RBS38-1, was taken just below the first one, from depths of 5 to 10 cm. The collected samples were a finely dispersed coal-bearing rock. The ground temperature at the sampling site was 58 °C.

For sample RBS38, the anion content of the aqueous extract was determined. The dominant anion was nitrate (184.3 mg/L), while sulfate (4.5 mg/L), fluoride (12.1 mg/L), and chloride (0.4 mg/L) were found in smaller amounts.

**Figure 2:**
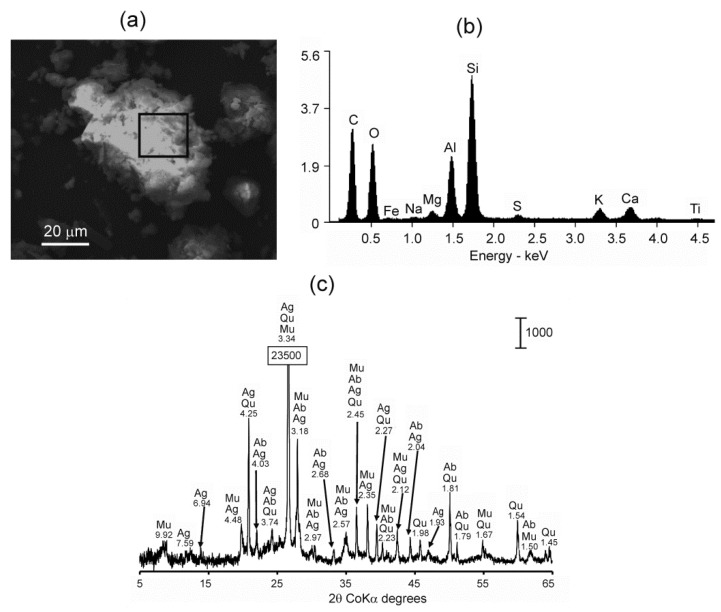
Characteristics of the chemical composition of the RBS38 sample. Scanning electron micrograph (**a**), the corresponding EDS spectrum (**b**), and the X-ray diffractogram showing the mineralogical composition (**c**). The vertical bar in (**b**) shows the scale of relative counts. Letter codes: *Qu*—quartz, SiO_2_, *Ag*—alunogen, Al_2_(SO_4_)_3_·17H_2_O, *Ab*—albite, NaAlSi_3_O_8_, *Mu*—muscovite, H_2_KAl_3_(SiO_4_)_3_.

The solid phase observed in the RBS38 sample by XRD contained various aluminosilicates (Figure 2). The detected alunogene, Al_2_(SO_4_)_3_·17H_2_O, is a typical product of coal fire discovered in larger quantities in burning coal mining waste dumps or as deposits derived from gases exhaled from surface vents associated with underground coal fires [19]. The scanning electron microscopy with microprobe elemental analysis of the sample confirmed aluminum and silicon presence and revealed noticeable amount of iron and titanium (Figure 2). Sulfur has also been detected by elemental analysis, which is consistent with the presence of alunogene.

### 3.2. Microbial Community Structure Revealed by 16S rRNA Profiling

**Figure 3:**
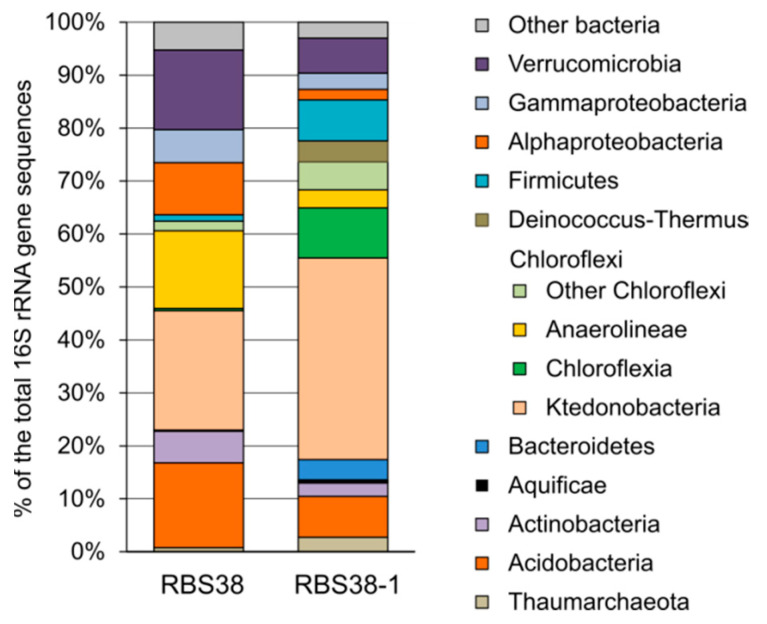
The relative abundance of taxonomic groups of microorganisms according to 16S rRNA gene profiling. “Other bacteria” comprises all reads not assigned to mentioned phyla (details are provided in Supplementary Table S1).

To characterize the compositions of microbial communities in samples RBS38 and RBS38-1, 24,094 and 44,336 sequences of 16S rRNA gene fragments were determined, respectively. As a result of clustering the obtained sequences, 92 OTUs were identified at the level of 97% identity. Alpha diversity indices indicate a lower diversity of the microbial community of the upper horizon RBS38 compared to the deeper ground layer, RBS38-1 (Table 1). The results of the taxonomic classification of the OTUs are shown in Figure 3 and Supplemental Table S1.

The pool of 16S rRNA gene reads was dominated by sequences of bacterial origin. The relative abundancies of archaea were 0.79% and 2.74% (hereinafter, of the total 16S rRNA gene sequences) in samples RBS38 and RBS38-1, respectively. The archaeal population was represented by two OTUs assigned to the candidate genera *Candidatus* Nitrososphaera and *Candidatus* Nitrosocaldus. Ca. Nitrososphaera predominated in the top layer, while Ca. Nitrosocaldus—in the deeper layer. Members of both genera are chemolithoautotrophs, receiving energy through aerobic oxidation of ammonium to nitrite; thermophiles were found among them [20,21,22].

Among Bacteria, members of the phylum Chloroflexi dominated and constituted 39.43% and 56.24% of the community in samples RBS38 and RBS38-1, respectively. More than half of Chloroflexi belonged to the class *Ktedonobacteria*, whose representatives were found both in common soils [23] and in terrestrial thermal ecosystems [24,25]. In particular, *Ktedonobacteria* were among the dominant groups in ecosystems with increased CO_2_ concentrations, such as fluids and soils in areas of naturally occurring CO_2_ gas vents [26,27,28].

**Figure 4:**
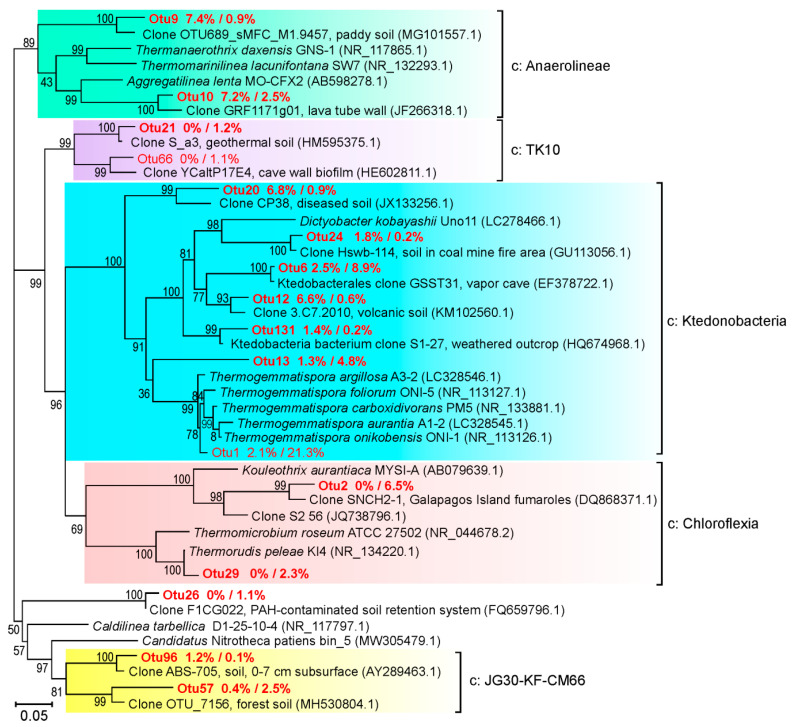
Maximum likelihood 16S rRNA gene phylogenetic tree of the phylum Chloroflexi. OTUs obtained in this work and assigned to the *Chloroflexi* are highlighted in red; only the ones accounting for >1% of reads in at least one of the two samples are shown. The shares of OTUs (in RBS38/RBS38-1) are shown after the OTU numbers. GenBank accession numbers are shown in parentheses after the clone or isolate names. The scale bar represents substitutions per nucleotide base.

A GenBank search revealed 16S rRNA sequences closely related to detected Chloroflexi OTUs in volcanic soils in Mexico (GenBank KM102560) and in the area of underground coal burning in China [7]. A single OTU comprising about 21% of the community in sample RBS38-1 was assigned to the genus *Thermogemmatispora* found in various terrestrial thermal ecosystems [29] (Figure 4). Many members of *Ktedonobacteria*, in particular *Thermogemmatispora carboxidivorans*, possess type 1 CO-dehydrogenase genes and are capable of oxidizing carbon monoxide [30,31]. The presence of an almost complete set of genes for the reductive tricarboxylic acids cycle indicates the possibility of autotrophic carbon fixation [31]. Some species of *Ktedonobacteria*, in particular *Thermogemmatispora* sp. T81 can aerobically oxidize trace amounts of hydrogen present in the atmospheric air [32].

In sample RBS38, about 15% of the community belonged to representatives of *Anaerolineae*, phylogenetically distant from the cultured species of this class (Figure 4). Most of the cultivated *Anaerolinea* are heterotrophs that grow on various carbohydrates, carrying out fermentation, aerobic or anaerobic respiration [33]. In sample RBS38-1, the relative abundance of *Anaerolineae* was only 3.4%. Members of the class *Chloroflexia* were present in significant amounts in the deeper sample (9.5%), but were nearly absent in RBS38 (0.4%). Among them, *Roseiflexaceae,* closely related to uncultivated lineages detected in fumaroles in the Hawaiian (KM278276) and Galapagos Islands [34], were the most numerous (6.5% in RBS38-1). The RBS38-1 community also harboured *Thermorudis peleae* (2.3%, Figure 4), a thermophilic CO-oxidizing bacterium isolated from geothermally heated soil in Hawaii [30].

The phylum Firmicutes accounted for 1.25% of the community in the RBS38 and 7.8% in the RBS38-1 sample. Most of them belonged to the genus *Tumebacillus*, whose representatives are aerobic heterotrophs found in soils and freshwater environments [35,36]. *Kyrpidia tusciae*, aerobic thermophilic facultative chemolithoautotrophic bacteria capable of oxidizing molecular hydrogen [37], was found in small amounts (0.23% in RBS38 and 0.78% in RBS38-1). *K. tusciae* were previously detected in the ground in the area of underground brown coal fire in Altai Mountains [38]. *Candidatus* Carbobacillus altaicus [38], another hydrogen-oxidizing thermophilic Firmicutes occurring in areas of underground coal fires, were also found but in minor amounts.

Two groups of thermophiles were found only in the deeper sample, RBS38-1. These are bacteria of the genus *Meiothermus* (phylum Deinococcus-Thermus), moderately thermophilic aerobic chemoorganoheterotrophs, previously found in geothermally heated soils [39]. Some members of this genus are also capable of reducing nitrate [40]. The share of *Meiothermus* in the RBS38-1 community was about 4%. About 0.6% of the community was represented by *Hydrogenobacter* sp. (phylum Aquificae), aerobic hydrogen-oxidizing lithoautotrophs usually isolated from hot springs [41]. One OTU assigned to the genus *Thermoflavifilum* (phylum Bacteriodetes) accounted for 2.79% of the community in the RBS38-1 sample, and 0.16% in the RBS38. The only cultured species of this genus, *Thermoflavifilum aggregans*, is an aerobic chemoorganotroph with a temperature optimum of growth of about 60 °C [42].

A single OTU phylogenetically close to *Nitrospira calida* (97.2% identity of 16S rRNA sequences) accounted for about 0.6% of the microbiome in both RBS38 and RBS38-1 samples. This moderately thermophilic nitrite-oxidizing bacterium of the phylum Nitrospirae was isolated from a geothermal spring [43]. *Nitrospira* sp. can complete nitrification, started by the *Thaumarchaeota*, and form nitrate, which was found in the aqueous extract.

In the upper layer of the ground, members of the phyla Acidobacteria (16.0%), Verrucomicrobia (15.0%), Actinobacteria (6.0%), Planctomycetes (1.0%), as well as Proteobacteria of the classes alpha (9.3%) and gamma (6.2%) were abundant. In the deeper sample RBS38-1, almost all OTUs related to these lineages were also present, but their relative abundancies were several times smaller than in RBS38. The statistical significance of these differences cannot be assessed, because we did not analyse replicated subsamples. A notable exception is gamma-proteobacteria of the family *Methylococcaceae*, whose share in RBS38 was 0.77%, and 1.84% in RBS38-1. The cultivated members of this family are aerobic methanotrophs [44] and, probably, the growth of this group is supported by methane coming from the coal bed.

The phylum Verrucomicrobia was dominated by a single OTU assigned to the candidate genus *Candidatus* Udaeobacter, whose share in the community was 14.9% in the upper ground layer and 6.6% in the lower one. Ca. Udaeobacter is ubiquitous and consistently one of the most abundant soil bacterial phylotypes worldwide, particularly in grasslands [45]. Genomic analysis showed that Ca. Udaeobacter is an aerobic heterotroph with numerous amino acid and vitamin auxotrophies, which probably obtains nutrients from dead microbial biomass [46]. At the same time, this bacterium is capable to oxidize the atmospheric trace gas H_2_ to generate energy [46]. It is possible that the hydrogen arriving with the released coal gases stimulates the growth of Ca. Udaeobacter in the upper horizons of the ground, while the rise of temperature with depth limits its growth in deeper layers.

### 3.3. Comparison with Other Coal Fire Sites

Thermal ecosystems associated with underground coal burning sites are less common and much less studied than geothermal features such as hot springs and other ecosystems associated with volcanic activity. The first studies on the composition of soil microbial communities in areas of underground coal fires were carried out in Centralia (Pennsylvania, USA) [9,47]. The 16S rRNA gene profiling studies revealed the presence of archaea (Crenarchaeota), as well as bacteria of the phyla Chloroflexi (the family *Thermogemmatisporaceae* of the order *Ktedonobacterales*), Acidobacteria, Proteobacteria, Parvarchaeota, Bacteroidetes, Elusimicrobia, and Gemmatimonadetes. In subsequent metagenomics studies, it was shown that microorganisms with a smaller cell and genome size predominate in areas of soil with a higher temperature [48]. For example, metagenome-assembled genomes of the Ca. Udaeobacter from hot soils turned out to be smaller in size than Ca. Udaeobacter genomes from ordinary soils. This microorganism was also found in the samples we examined.

Microbial communities of heated soils around coal-fire gas vents in Xinjiang, China, were investigated using T-RFLP analysis and 16S rRNA gene clone libraries [7]. Among the dominant groups of microorganisms, representatives of the phyla Firmicutes, Proteobacteria, Acidobacteria, Bacteroidetes, Planctomycetes and Actinobacteria were found; archaea have also been detected. The most numerous group were Firmicutes (71.4% of all 16S rRNA clones), mainly members of the genera *Bacillus* and *Paenibacillus*. Ammonium-oxidizing Thaumarchaeota were found among Archaea.

The previously studied microbial community of the heated ground in the area of underground coal fire in Altai Mountains was the simplest in composition [38]. It included three dominant phylotypes, all of which belonged to the phylum Firmicutes. It was an aerobic heterotroph Ca. Carbobacillus altaicus, anaerobic chemolithoautotroph *Brockia lithotrophica*, and aerobic bacterium *Hydrogenibacillus schlegelii*, capable of both using organic compounds and growing autotrophically. *K. tusciae* has also been found in these samples. All these bacteria can receive energy through the oxidation of molecular hydrogen, which probably supports the growth of this community [38].

The microbial community studied in our work turned out to be much more taxonomically diverse and included, along with probable thermophiles, and typical soil heterotrophic microorganisms, especially in the upper ground layer. Since areas of the terrain where underground coal fire was observed are adjacent to ordinary soil with rich vegetation, the ingress of wind-carried soil particles can provide an influx of organic substrates and soil microorganisms, which was not observed in the area of underground coal burning in the Altai Mountains site [38]. Microorganisms typical of soils were also found in samples from Pennsylvania [9,47,48] and China [7].

Hydrogenotrophic Firmicutes detected in the area of underground coal fire in the Altai Mountains were also found in the samples studied in the present work (*Ca*. Carbobacillus altaicus and *K. tusciae*), but in minor amounts (<1%). However, other microorganisms presumably capable of oxidizing the high-energy components of coal gases, hydrogen and CO, formed a significant part of the communities. The dominant microbial groups included Chloroflexi, phylogenetically close to thermophilic hydrogen- and CO-oxidizing species. Among the discovered thermophiles, members of the genus *Hydrogenobacter* (phylum Aquificae) probably possess the ability to oxidize hydrogen. Another major group of probable hydrogenotrophs was Verrucomicrobia of the genus Ca. Udaeobacter. Although Ca. Udaeobacter has not been previously reported in thermal ecosystems, the abundance of this lineage suggests that these bacteria are functionally active “natural” components of the studied ecosystem rather than contaminants associated with soil particles brought from the surrounding areas.

In contrast to the site of underground coal fire in the Altai Mountains, here a significant part of the community was made up of phylotypes, whose cultivated relatives are thermophiles capable of CO oxidation under aerobic conditions. These are, first of all, members of the *Ktedonobacteria*, accounting for more than a third of the community in RBS38-1. Although we were unable to collect gas samples for composition analysis, it should be noted that in the coal gasification reaction (3 C + O_2_ + H_2_O → H_2_ + 3 CO), 3 CO molecules are formed per hydrogen molecule; i.e., it is likely that the coal gases contained significant amounts of carbon monoxide.

Thermal ecosystems associated with areas of underground coal burning emerged only tens to hundreds of years ago and are young compared to geothermal objects. It was proposed that thermophilic Firmicutes, whose spores can spread over long distances, may be the first “colonizers” of such newly formed thermal ecological niches [38]. It is possible that other thermophiles could have entered the object under study in a similar way. In particular, members of *Ktedonobacteria* can form cells that structurally resemble spores, in which they are able to survive a lack of nutrients or harsh environmental conditions [31].

## 4. Conclusions

Microbial communities were dominated by members of the class *Ktedonobacteria* known to be capable of oxidizing hydrogen and carbon monoxide. Another abundant lineage, Ca. Udaeobacter, can generate energy through the oxidation of hydrogen. Thermophilic hydrogenotrophic Firmicutes constituted a minor part of the community. Overall the results obtained suggested that main components of coal gases, hydrogen and carbon monoxide, provides electron donors for microbial growth. Reaching the surface, these high energy compounds are exposed to atmospheric oxygen, which provides conditions for the development of aerobic hydrogen and CO oxidizing microorganisms.

## Figures and Tables

**Table 1:** Diversity of prokaryotes in microbial communities of RBS38 and RBS38-1.

Parameter	RBS38	RBS38-1
OTU number	67	92
Chao1 index	67.8	92.5
Shannon_e index	3.36	3.21
Simpson index	0.0534	0.0763

## Data Availability

All sequences associated with this work have been deposited at the National Center for Biotechnology Information under BioProject ID: PRJNA697856.

## Supplementary Materials

The following are available online at https://www.mdpi.com/article/10.3390/microorganisms9050948/s1, Table S1: Relative abundance and taxonomic classification of OTUs.
